# Genomic instability genes in lung and colon adenocarcinoma indicate organ specificity of transcriptomic impact on Copy Number Alterations

**DOI:** 10.1038/s41598-022-15692-8

**Published:** 2022-07-11

**Authors:** Chinthalapally V. Rao, Chao Xu, Yuting Zhang, Adam S. Asch, Hiroshi Y. Yamada

**Affiliations:** 1 Department of Medicine, Hematology/Oncology Section, Center for Cancer Prevention and Drug Development, University of Oklahoma Health Sciences Center (OUHSC), Oklahoma City, OK USA; 2 Hudson College of Public Health, University of Oklahoma Health Sciences Center (OUHSC), Oklahoma City, OK USA; 3 Stephenson Cancer Center, University of Oklahoma Health Sciences Center (OUHSC), Oklahoma City, OK USA; 4 Present Address: 975 NE 10th St., BRC1203, Oklahoma City, OK 73104 USA; 5 Present Address: 801 Northeast 13th Street, Room 321, P.O. Box 26901, Oklahoma City, OK 73190 USA; 6 Present Address: 975 NE 10th St. BRC1209, Oklahoma City, OK 73104 USA; 7 Present Address: 800 NE 10th St., 6th Floor, Oklahoma City, OK 73104 USA; 8 Present Address: 975 NE 10th St., BRC1207, Oklahoma City, OK 73104 USA

**Keywords:** Cancer, Computational biology and bioinformatics, Drug discovery, Medical research, Oncology

## Abstract

Genomic instability (GI) in cancer facilitates cancer evolution and is an exploitable target for therapy purposes. However, specific genes involved in cancer GI remain elusive. Causal genes for GI via expressions have not been comprehensively identified in colorectal cancers (CRCs). To fill the gap in knowledge, we developed a data mining strategy (Gene Expression to Copy Number Alterations; “GE-CNA”). Here we applied the GE-CNA approach to 592 TCGA CRC datasets, and identified 500 genes whose expression levels associate with CNA. Among these, 18 were survival-critical (i.e., expression levels correlate with significant differences in patients’ survival). Comparison with previous results indicated striking differences between lung adenocarcinoma and CRC: (a) less involvement of overexpression of mitotic genes in generating genomic instability in the colon and (b) the presence of CNA-suppressing pathways, including immune-surveillance, was only partly similar to those in the lung. Following 13 genes (TIGD6, TMED6, APOBEC3D, EP400NL, B3GNT4, ZNF683, FOXD4, FOXD4L1, PKIB, DDB2, MT1G, CLCN3, CAPS) were evaluated as potential drug development targets (hazard ratio [> 1.3 or < 0.5]). Identification of specific CRC genomic instability genes enables researchers to develop GI targeting approach. The new results suggest that the “targeting genomic instability and/or aneuploidy” approach must be tailored for specific organs.

## Introduction

Genomic instability in cancer affects cancer development and evolution, causing drug resistance and poor prognosis, thus impacting therapy outcomes in clinic^1–3^. Hence, the “targeting genomic instability and/or aneuploidy for cancer therapy” concept has been proposed^4^. For contemporary targeted drug development, genomics information is critical^5^. Although some signatures for genomic instability in select organs were identified [e.g.,^6^], genes involved in genomic instability in cancer have been elusive, preventing researchers from designing specific agents for targeted therapies. Gene expression analysis of pan-cancer datasets indicated that mitotic signature increases and immune signature decreases were characteristics of high CNA cancers^7^, suggesting the roles of mitotic mis-regulation in generating CNA and of immune functions in antagonizing cancer cells with CNA. Although the notion of immunosurveillance of genomic instability and aneuploidy has long been proposed, few involved genes have been identified and the molecular mechanisms remain to be determined^8,9^.

Results with transgenic mouse models from our and other laboratories have indicated dual effects of genomic instability in the body on cancer, for both tumor suppression and oncogenesis^10,11^. Mitosis-targeting genomic instability models (Chromosome instability [CIN] models; e.g., Mad2, BubR1, Sgo1) have demonstrated the role of genomic instability as a disease modifier, resulting in tumor proneness in organs including the colon, lung, and liver later in life^12–17^. Although genomic instability is prevalent in most solid tumors, based on the tumor profile in genomic instability transgenic mice, we hypothesized that genomic instability has prominent effects for cancer development and/or disease modification in the colon, liver, and lung^18^. To identify specific genes involved in genomic instability in human lung adenocarcinoma, we developed a novel data mining strategy, GE-CNA, which is an approach to identify all genes whose expression associates with increased or decreased tumor CNA^18^. Pathway analysis revealed that (a) amplification/insertion CNA is facilitated by over-expressions of DNA replication stressors and suppressed by a broad range of immune cells (T-, B-, NK-cells, leukocytes), and (b) deletion CNA is facilitated by over-expressions of mitotic regulator genes and suppressed predominantly by leukocytes guided by leukocyte extravasation signaling. Among the 39 CNA- and survival-associated genes, purine metabolism (PPAT, PAICS), immune-regulating CD4-LCK-MEC2C and CCL14-CCR1 axes, and ALOX5 emerged as survival-critical pathways. These pathways/genes are potential therapy drug targets for lung adenocarcinoma^18^.

With the lung cancer results, we continued the GE-CNA analysis with cancers in liver and colon, anticipating similar gene profile, thus common genes for targeting genomic instability, would emerge. As naturally-occurring polyploidization in liver complicating the CNA datasets and analysis, we focused on colon cancer. In the United States, colorectal cancer (CRC) is expected to cause about 52,580 deaths during 2022, and is the second most common cause of cancer deaths when cancer deaths for men and women are combined^19^. Thus, CRCs remain a major target for prevention and therapy development. In CRCs, tumor development is associated with progressive mutational accumulation, as indicated in the “Vogelgram”^20^. Functional analysis of the frequently mutated genes indicated that each of the mutations in the gene (e.g., APC, TP53, FBXW7/hCDC4, PI3K-PTEN, K-RAS) can cause genomic instability, directly or indirectly^21^. Thus, a part of genomic instability in CRCs is linked to mutations in key oncogenic/tumor-suppressing genes. In addition, epigenetic modulations, environmental challenges from microbiota, and transcriptomic and microRNA changes, which are also suggested to affect genomic instability, were reported [e.g.,^22–26^]. Among these events impacting genomic instability, transcriptomic alterations, especially over-expressions, are most feasible to manipulate with drugs, while restoring mutated genes is technically difficult. However, transcriptomic alterations associated with genomic instability in CRCs have not been comprehensively identified, and our understanding of the impact of the transcriptomic landscape on genomic instability in CRCs remains incomplete. Hence, we set out to apply the GE-CNA data mining approach to identify genes and pathways involved in genomic instability in CRCs via transcriptomic mis-regulations.

## Materials and methods

### GE-CNA analysis

We downloaded the Colorectal Adenocarcinoma (TCGA, PanCancer Atlas, 2018) datasets from cBioportal (https://www.cbioportal.org/study/summary?id=coadread_tcga_pan_can_atlas_2018)^27,28^, a publicly available database. All following methods were carried out in accordance with relevant guidelines and regulations. The datasets included survival and clinical data for 594 patients. Among these patients, we also collected the available the gene expression profile and copy number alterations of 592 patients, and whole exome sequencing (WES) mutation profile of 528 patients. The batch normalized gene expression Z-scores by RSEM^29^ from Illumina HiS-eq_RNASeqV2 were used. The downloaded copy-number alteration (CNA) was estimated by GISTIC 2.0^30^. Neutral or no change CNA was indicated by 0. Gain/amplification CNA was indicated by a positive value, while a negative value indicated deletion CNA. Amplification CNAs and deletion CNAs were analyzed jointly and separately.

In the gene expression file, we had 20,471 genes of 592 subjects. We excluded 3073 genes that were missing in more than 1/3 of subjects. The included genes were complete in all subjects. We sorted each gene by its expression in all subjects and selected the top 10 and bottom 10 subjects. The selected subjects were assigned to a high expression group and a low expression group, accordingly. Next, we extracted the subjects’ CNA counts in the high and low expression groups from the CNA file. Student’s *t*-test was used to examine the difference in CNA counts in the high group vs. the low group. Multiple-testing was corrected by *q*-value^31^. The significance level was 0.05.

Further, we divided the significant genes into two groups: higher expression that resulted in more CNAs and higher expression that resulted in fewer CNAs. We employed the bioinformatics tool IPA (Ingenuity Pathway Analysis, QIAGEN, Inc., https://www.qiagenbioinformatics.com/products/ingenuity-pathway-analysis) to conduct the gene set enrichment analyses^32^. The Benjamini–Hochberg corrected *p*-value^33^ provided by IPA was reported and evaluated at the significance level of 0.05. Also, we presented the pathway graphs from IPA.

The survival analysis of the gene alteration with regard to the overall survival was examined by the Cox Proportional-Hazards (CoxPH) Model. Age and tumor stage were adjusted as covariates, which were selected by their univariate CoxPH analysis *p*-value < 0.05. All available variables, such as age, sex, race, and tumor stage, were considered. The race groups with small numbers of patients were combined. The race variable analyzed in CoxPH model had two levels: White and Other. The sub-levels of tumor stage under each stage of stages 1 to 4 were combined, which resulted in four levels used in the analysis. We excluded patients with incomplete data. The Hazard Ratio (HR) and p-value of the gene were reported. The definitions of “altered” and “unaltered” subjects were from cBioportal. Briefly, an altered subject was a subject having any type of high-level CNA amplification, CNA homozygous deletion, or WES mutation. Otherwise, a subject was considered an unaltered subject. We compared the difference in gene expression levels in the altered and unaltered groups using the Wilcoxon rank sum test. The significance level was 0.05. We presented the survival curves and boxplots by altered/unaltered group. We implemented all statistical analyses using R (v4.0.3) and R packages.

The major reason to only use extreme high and low gene expression groups is to increase the statistical power by enriching the presence and increasing the effect size of the causal genetic factors. 592 is not a large sample size to separate, thus we use all samples to maximize the study power.

To estimate the magnitude of HR, we employed the following categories: small (not trivial, but possibly inconsequential), medium (likely consequential), and large (very likely consequential) HRs comparing 2 groups would be approximately 1.3, 1.9, and 2.8, respectively^34^.

### Availability of data and materials

We obtained original tumor data from the cBioportal (https://www.cbioportal.org/study/summary?id=coadread_tcga_pan_can_atlas_2018)^27,28^, which is a publicly available database. The data were openly available for download. Main data generated or analyzed during this study are included in this published article and its supplementary information files. All the datasets used and/or analyzed during the current study will be available from the corresponding author on reasonable request.

## Results

**Figure 1:**
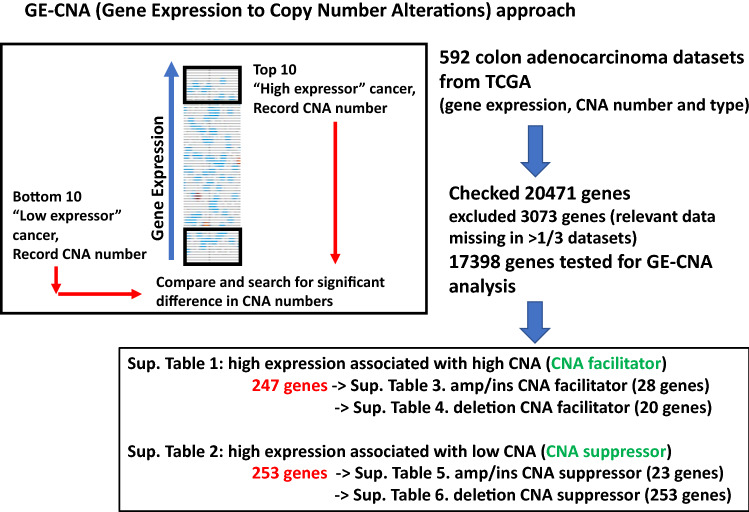
Identifying genes associated with Copy Number Alterations in colon adenocarcinoma with the “Gene Expression to Copy Number Alterations” (“GE-CNA”) approach. For all genes, we recorded CNA for high expressor tumors (*N* = 10) and for low expressor tumors (*N* = 10). The CNA from the “high expressor” and “low expressor” groups were compared using unpaired *t*-test for each gene, testing the correlation between gene expression and numbers of CNA (*q*-value < 0.05). Genes whose high expression was associated with high CNA were annotated as CNA suppressors, while genes whose high expression was associated with low CNA were annotated as CNA suppressors. Genes specifically associated with a type of CNA ([a] amplification/insertion [amp/ins] CNA, often associated with Microsatellite Instability [MIN], and [b] deletion CNA, often associated with mitotic error-mediated Chromosome Instability [CIN]), were identified. Figure was generated with cBioportal (https://www.cbioportal.org/datasets).

We applied GE-CNA to 592 CRCs in the TCGA database (Fig. 1). Supplementary Table 1 shows 247 genes whose high expression associates with high tumor CNA, and thus are annotated as CNA facilitators. Functional denotation and pathway analysis indicated that (i) the genes are functionally diverse and (ii) there was no statistically significant enrichment (corrected *P* < 0.05) of a specific pathway. The lack of specific enrichment is a major difference from the previous results from lung adenocarcinoma that showed enrichment on mitotic regulators and DNA replication pathways^18^.

**Figure 2:**
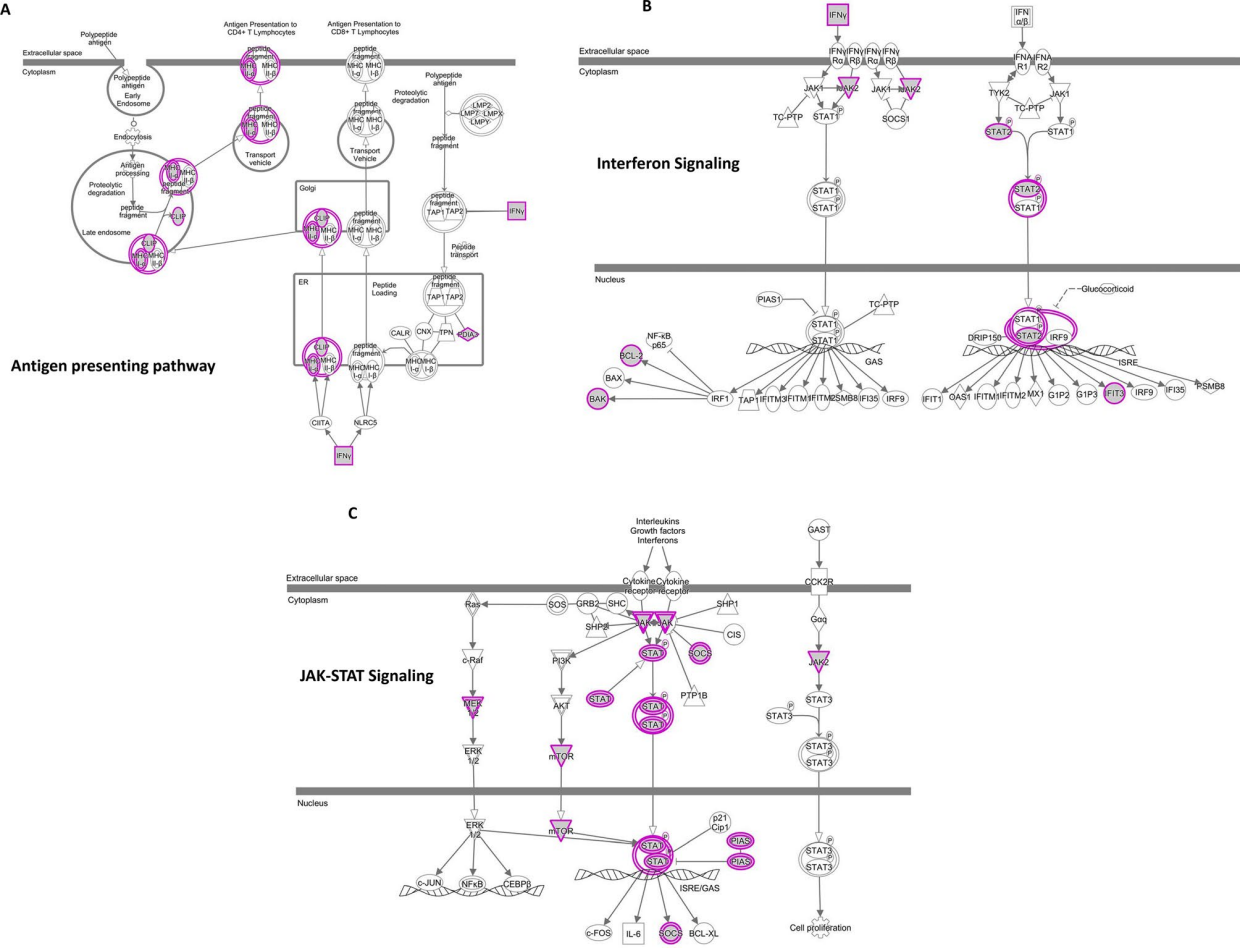
Pathway analysis of CNA suppressors. The 247 CNA facilitator genes in Supplementary Table 1 did not show significant enrichment in a pathway. The 253 CNA suppressor genes in Supplementary Table 2 were further subcategorized to amplification/insertion CNA suppressors (Supplementary Table 5) and deletion CNA suppressors (Supplementary Table 6). Amp/ins CNA suppressors include only 23 genes, while deletion CNA suppressors include 253 genes, suggesting that CRC cells with amplification/insertion CNA and deletion CNA are suppressed through different modalities. Deletion CNA suppressor genes show enrichment in the (**A**) Antigen Presentation Pathway, (**B**) Interferon signaling pathway, and (**C**) JAK-STAT signaling pathway, suggesting that CRC cells carrying CIN-associated deletion CNA are targeted by these immune-associated pathways and that they represent an immunosurveillance mechanism of CIN cells in CRC. Purple highlighting indicates particular genes with significant GE-CNA correlations and/or a cluster of such genes in the IPA pathways. Figures were generated with IPA (Ingenuity Pathway Analysis, QIAGEN, Inc., https://www.qiagenbioinformatics.com/products/ingenuity-pathway-analysis).

Supplementary Table 2 shows 253 genes whose high expression associates with low tumor CNA, and thus are annotated as CNA suppressors. The enriched pathways (corrected *P* < 0.05) were: Interferon Signaling (BAK1, BCL2, IFIT3, IFNG, JAK2, STAT2), Antigen Presentation Pathway (CLIP, MHC II-alpha), Heme Biosynthesis II (ALAS1, CPOX, FECH), Natural Killer Cell Signaling (HSPA5, IFNG, IL15, JAK2, KIR2DL4, MAP2K1, MTOR, NCR1, ULBP3), Retinoic acid Mediated Apoptosis Signaling (TRAIL-R, PARP), JAK/Stat Signaling (JAK2, MAP2K1, MTOR, PIAS2, SOCS6, STAT2), Glucocorticoid Receptor Signaling (HSP90, HSP70, NCOR, TFIIA, OXPHOS), Heme Biosynthesis from Uroporphyrinogen-III I (CPOX, FECH), and Glutathione Redox Reactions II (GSR, PDIA3) (Fig. 2. pathway analysis of CNA suppressors). The functions of the pathways are (i) immune function and its regulation (Interferon signaling, Antigen Presentation, Natural Killer cell signaling); (ii) growth signaling (JAK/STAT, Glucocorticoid receptor); (iii) apoptosis (Retinoic acid); (iv) Heme biosynthesis II (ALAS1, CPOX, FECH); and (v) Glutathione redox signaling.

To obtain further mechanistic insight on CNA generation/suppression in CRC, we questioned whether amplification/insertion CNA and deletion CNA are differentially affected by different sets of genes. In lung adenocarcinoma, amplification/insertion CNA was facilitated by 161 genes whose main functions are involved in the DNA replication and repair pathways, suggesting that amplification/insertion CNA is predominantly driven by MIN or CIN caused by DNA replication stress^18^. In contrast, deletion CNA was associated with 187 genes that were enriched with known mitotic regulators, suggesting a link between mitotic errors and deletion CNA in lung adenocarcinoma. In CRCs, we identified 28 genes associated with amplification/insertion CNA increases (Amp/ins CNA facilitators; Supplementary Table 3), and 20 genes associated with deletion CNA increases (Deletion CNA facilitators; Supplementary Table 4). The number of identified genes is several-fold fewer than those in the lung, and the genes were not significantly concentrated in particular pathways, nor were the same genes identified in lung adenocarcinoma, indicating organ specificity in the profile. Yet, there are limited similarities; a few of the genes in Supplementary Table 3 and 4 are indeed involved in DNA metabolism and/or mismatch repair. For example, ASTE1/HT001 encodes a nuclease associated with MIN^35–37^. Recently, ASTE1 was identified as a downstream effector of the shieldin complex and a structure-specific DNA endonuclease that specifically cleaves single-stranded DNA and 3′ overhang DNA^38^. DNASE1 encodes Deoxyribonuclease1, which may be involved in clearance of cell-free DNA that serves as circulating tumor marker as well as playing a role in SLE pathogenesis^39^. Genes involved in RNA metabolism are also noted. DDX27 encodes a putative RNA helicase. PRPF6 encodes pre-mRNA processing factor 6. RPS6KA6 encodes ribosomal protein S6 kinase A6, a kinase downstream to the ERK/MAPK pathway, and is being investigated as an inhibition target for various cancers ^40^. SMG5 encodes SMG5 nonsense-mediated mRNA decay factor, which is thought to provide a link to the mRNA degradation machinery involving exonucleolytic pathways ^41^. Therefore, nucleic acid metabolism emerged as a factor affecting CNA in CRC.

The CNA suppressor genes in Supplementary Table 2 were further subcategorized to amplification/insertion CNA suppressors (Supplementary Table 5) and deletion CNA suppressors (Supplementary Table 6). Supplementary Table 5 includes only 23 genes, and Supplementary Table 6 includes 253 genes, suggesting that CRC cells with amplification/insertion CNA and deletion CNA may be suppressed through different modalities, which agrees with results from lung adenocarcinoma. Pathway analysis indicated that (a) amplification/insertion CNA suppressor genes show enrichment in Maturity Onset Diabetes of Young (MODY) Signaling (FABP2, GAPDH), NADH Repair (GAPDH), and Heme Biosynthesis from Uroporphyrinogen-III I (FECH) pathways; and (b) deletion CNA suppressor genes show enrichment in Antigen Presentation Pathway (Fig. 2A), Interferon Signaling (Fig. 2B), Heme Biosynthesis II, Natural Killer Cell Signaling, Retinoic acid Mediated Apoptosis Signaling, JAK/Stat Signaling (Fig. 2C), Glucocorticoid Receptor Signaling, Heme Biosynthesis from Uroporphyrinogen-III I, and Glutathione Redox Reactions II pathways. The enrichment profiles suggest that cells with amplification/insertion CNA are suppressed with metabolic modulations, while cells with deletion CNA are targeted by immune cells and/or by growth and cell death-related signaling, also affected by redox signaling.

**Figure 3:**
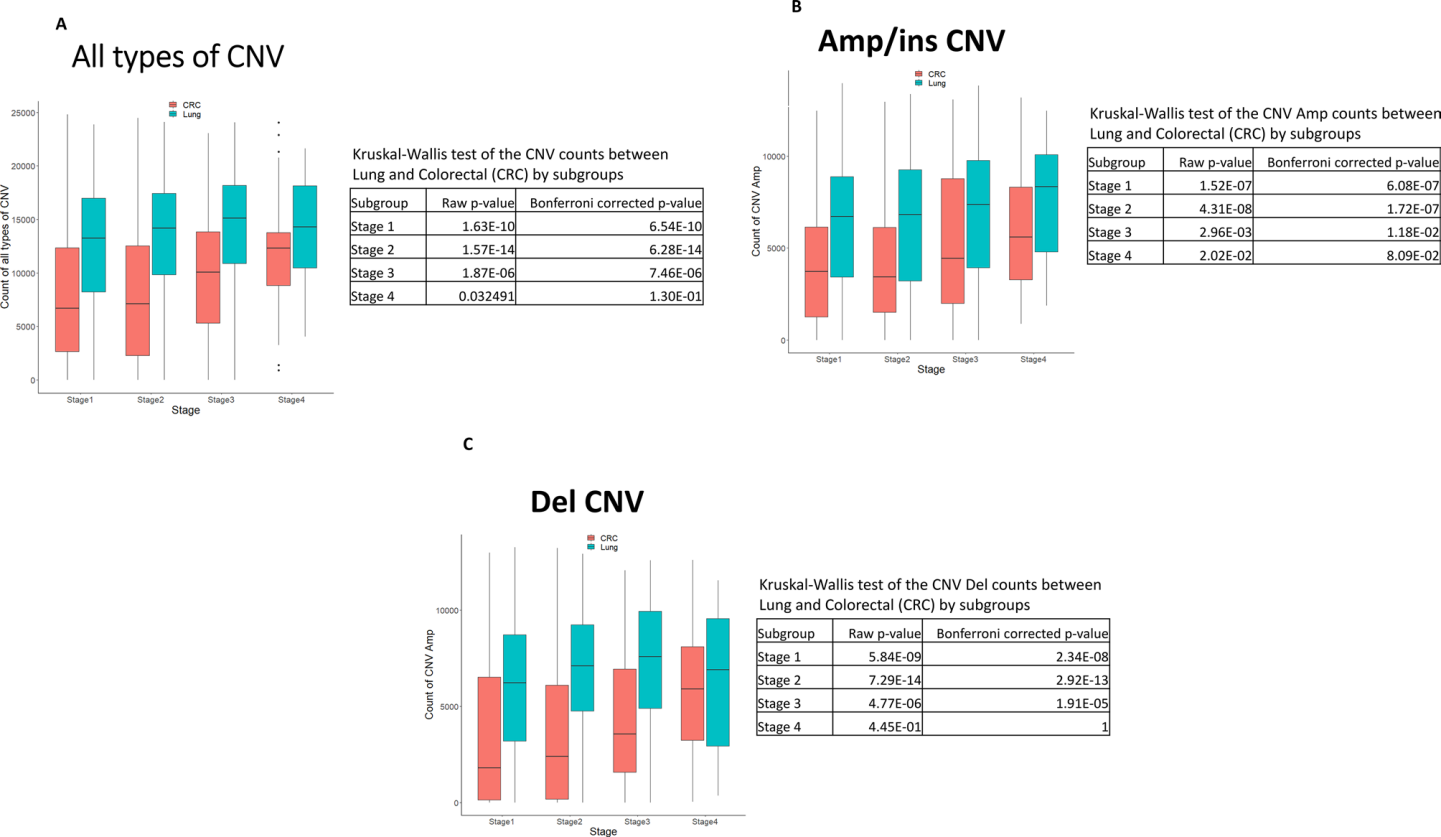
Lung adenocarcinomas carry higher CNA than do CRCs at all stages and in both types of CNA (amp/ins CNA and deletion CNA). (**A**) At all stages, lung adenocarcinomas carry higher numbers of CNA (all types of CNA) than do CRCs (green: lung adenocarcinoma, orange: CRC). The difference is particularly notable at earlier stages. For stages 1–3, the difference was statistically significant (Bonferroni corrected *p*-value < 0.05). The trend is the same in both (**B**) Amp/ins CNA and (**C**) deletion CNA. Figures were generated from R v4.0.3 (https://www.R-project.org/).

The notable differences in pathway profiling results between lung adenocarcinoma and CRC led us to hypothesize that the total number of CNA is different between lung adenocarcinoma and CRC; one of the cancer types would show higher CNA. We compared total CNA numbers by cancer stages (Fig. 3A). In both cancers, cancer CNA increases over stages. In all types of CNA, in all stages, lung adenocarcinoma showed higher CNA than did CRC. The differences were significant in stages 1, 2, and 3 (corrected *P* < 0.05). Only in stage 4, due to an increase of CNA in CRC, did the gap in CNA numbers shrink to a non-significant level (Bonferroni corrected *p*-value = 0.13). The results were the same for amplification/insertion CNA (Fig. 3B) and for deletion CNA (Fig. 3C); CNA were consistently higher in lung adenocarcinoma than in CRC, regardless of the type. Based on the gene profile differences and CNA numbers between lung adenocarcinoma and CRC, we suspect that (a) major CNA generation mechanisms vary among cancers; (b) a transcriptome-driven mechanism is dominant in lung adenocarcinoma, while a mutation-driven mechanism is prominent in CRC; and (c) a transcriptome-driven mechanism of CNA generation is more aggressive than a mutation-driven mechanism.

The genes whose expression levels are associated with CNA are all potential targets to modulate genomic instability, which would affect therapy outcome. However, even if modulation of the gene expression can curtail genomic instability, if the modulation does not affect patients’ survival, the modulation approach would be futile. With this reasoning, we applied secondary screening, searching for genes whose expression levels are also significantly associated with survival rate of patients (*P* < 0.05). The secondary screening to identify genes whose expression levels were associated both with CNA and survival rate (i.e., “survival-critical”) yielded 11 genes from 247 CNA facilitators in Supplementary Table 1, and 16 genes from 253 CNA suppressors in Supplementary Table 2 (Table 1, Table 2). As indicated in Table 1, all the 27 select “survival-critical” genes showed significant differences in average CNA/CNV between high expressor and low expressor.

**Table 1:** Data for Gene Expression and Copy Number Alteration (GE-CNA) on initially-identified 27 “survival critical” genes.

	High expression group		Low expression group		*t*. test	
	Average # of CNVs	SD	Average # of CNVs	SD	*p* value	*p*. adjust. *q* value
**CNA facilitators**						
CAPS	14,892.8	4412.615	2347.6	2412.918	1.66E-06	0.001333
CCDC115	14,359.3	5092.752	3448.8	4751.292	0.000104	0.011347
ATP6AP1	12,126.8	4855.072	3390.3	3034.437	0.000218	0.017405
NBEAP1	14,700.2	4783.089	6138	3591.707	0.000311	0.021176
SPANXC	14,463	1873.704	6901.6	4500.857	0.000361	0.022546
TIGD6	14,767.4	4393.891	6187.2	4481.801	0.00041	0.023795
C7ORF13	13,171.1	5601.803	3521	4346.452	0.000484	0.025349
TMEM184A	12,130.2	2904.1	4049.2	4924.642	0.00048	0.025349
F8A1	14,166.3	4575.244	5996.1	4508.695	0.0008	0.032097
LZTS3	11,336.4	4583.305	3650	4075.844	0.000933	0.034692
OLMALINC	13,573.4	6549.111	4271.7	3132.232	0.00139	0.042547
**CNA suppressors**						
WARS	1849.9	2042.03	12,385.9	3529.064	8.72E-07	0.001118
FOXD4L1	3028.6	3274.165	13,484.1	3968.791	5.65E-06	0.002873
VWA5B2	5133	3112.157	12,829.7	2520.75	1.16E-05	0.004001
DDB2	3286.4	3690.682	14,951.7	5218.157	2.72E-05	0.006084
EPOR	3647	3455.342	11,305.9	2558.743	3.27E-05	0.00635
ROBO3	4185.4	3290.998	12,872.1	4243.073	8.69E-05	0.01051
PKIB	2970	4398.217	11,411.5	3568.829	0.000193	0.016055
TMED6	4789.4	4295.765	12,499.6	3371.77	0.000339	0.021989
APOBEC3D	2925.1	2451.516	13,097.9	6163.835	0.00042	0.023986
B3GNT4	5193.3	4830.085	13,538.8	3636.482	0.000437	0.024567
CLCN3	5522	3753.278	12,298.4	3620.061	0.00066	0.029537
FOXD4	4987	4658.925	12,645.9	3707.015	0.000789	0.032024
ZNF683	3835	4311.881	10,868.4	3498.788	0.000892	0.033785
EP400P1	3915.2	2512.353	12,900.2	6281.908	0.001276	0.040754
KLHDC7B	5436.4	5035.776	14,321.6	5600.079	0.001555	0.044346
MT1G	6955.3	4964.891	14,235.3	3544.305	0.001618	0.045402

The data on GE-CNA correlation (see Fig. 1 for details) for the select 27 genes. There are significant differences in CNVs (= CNAs) between high expressor and low expressor of the select 27 genes. Genes whose high expression is associated with higher CNV/CNA are annotated as CNA facilitators. Genes whose high expression is associated with lower CNV/CNA are annotated as CNA suppressors.

**Table 2:** List of 18 (27) survival critical genes.

Gene								Expression in altered group				Expression in unaltered group				test *p* value	
Survival-critical		Alterations	Drug needed	Entrez_id	Table	hr	*p* value	*n*	Mean	SD	Median	n	mean	SD	Median	Wilcox	t-test
**CNA facilitators**		Decrease correlates with															
CAPS	Calcyphosine, Ca2 + -binding, ion transport, V-ATPase assembly	Poor survival	Enhancer	828	4	1.907922041	0.375986	7	− 0.4095	0.183021	− 0.4771	585	0.15685	1.479923	− 0.2992	0.206713	6.88E-06
CCDC115	Coiled-coil domain-containing 115, ER, ion pump																
ATP6AP1	ATPaseH + transporting Accessory protein 1, vacuolar ATPase																
NBEAP1	Neurobeachin pseudogene 1																
SPANXC	SPANX family member C, testis-specific, metastasis/cancer antigen																
TIGD6	Tigger Transposable element derived 6, transposon, similar to cenpB	Better survival	**Inhibitor**	81,789	3	**0.455162325**	0.442975	11	− 0.35823	0.733154	− 0.5256	581	− 0.05557	1.20006	− 0.269	0.45003	0.208536
C7ORF13	Testis-specific, facilitates migration, EMT via mir																
TMEM184A	Transmembrane protein 184A, vesicle transport. Heparin receptor																
F8A1	Coagulation factor VIII associated 1, vesicular transport of early endosome																
LZTS3	Leucine zipper tumor suppressor family member 3, tumor suppressor																
OLMALINC	Oligodendrocyte maturation-associated long intergenic non-coding RNA																
**CNA suppressors**																	
WARS	Tryptophanyl-tRNA synthase1, damage-induced cytokine, immunomodulator	Poor survival	Enhancer	7453	5	**0.787757054**	0.814291	9	0.559444	1.566013	− 0.1172	583	− 0.15584	0.785742	− 0.3405	0.057173	0.208338
FOXD4L1	Forkhead Box D4 like 1, TF	Better survival	**inhibitor**	200,350	6	1.426462336	0.4651	21	0.637481	1.37034	0.1306	571	− 0.06577	0.898184	− 0.2856	0.002956	0.029834
VWA5B2	Von Willebrand factor A domain containing 5B2,	Poor survival	Enhancer	90,113	6	1.444342328	0.482426	22	− 0.07453	0.132804	− 0.1124	570	− 0.02217	0.902549	− 0.12405	0.144395	0.269443
DDB2	Damage specific RNA binding protein 2, UV damage repair, Xeroderma	Poor survival	Enhancer	1643	6	**2.85518E-06**	0.995514	4	0.78545	0.68143	0.60775	588	− 0.10943	0.937722	− 0.3888	0.02689	0.077632
EPOR	Erythropoietin receptor, JAK2-MAPK, PI3K, STAT signaling	Poor survival	Enhancer	2057	6	**0.912726465**	0.928492	7	− 0.02714	0.422443	0.1963	585	− 0.05042	0.908843	− 0.2833	0.362072	0.891349
ROBO3	Roundabout guidance receptor 3, migration, neurite outgrowth	Better survival	**Inhibitor**	64,221	6	1.058267165	0.888419	38	0.452355	1.426736	0.10805	554	− 0.08564	0.853085	− 0.34145	0.00036	0.027128
PKIB	cAMP-dependent PK inhibitor beta, <—> PKA, PI3K/AKT signaling	Better survival	**Inhibitor**	5570	6	1.468143772	0.597215	11	0.186236	1.018755	− 0.0177	581	− 0.02932	0.908974	− -0.3012	0.488256	0.501509
TMED6	Transmembrane p24 trafficking protein 6	Better survival	**Inhibitor**	146,456	6	**1.16204E-07**	0.994421	2	− 0.51305	0.20301	− 0.51305	590	− 0.02848	1.070154	− 0.3208	0.467364	0.157419
APOBEC3D	Apolipoprotein B mRNA editing enzyme catalytic subunit 3D, retrovirus inhibition	Better survival	**Inhibitor**	140,564	6	4.550147633	0.004547	12	0.790867	2.18234	0.0305	580	-0.14183	0.883672	-0.3564	0.101112	0.167277
B3GNT4	UDP glcNAc betaGal 1,3-N-acetylglucosaminyl transferase 4, Golgi, TM	Better survival	**Inhibitor**	79,369	6	2.35404515	0.103688	13	0.611408	1.947388	− 0.0383	579	− 0.02153	1.074378	− 0.3863	0.249043	0.265232
CLCN3	Chloride voltage-gated channel 3, endosomal protein trafficking, ion channel	Poor survival	Enhancer	1182	6	3.564447662	0.001042	29	− 0.53016	0.970466	− 0.4802	563	− 0.24354	1.005001	− 0.3456	0.168123	0.131658
FOXD4	Forkhead Box 4, TF, CRC progression	Better survival	**Inhibitor**	2298	6	1.788201318	0.220313	25	0.782232	1.357949	0.5783	567	-0.0695	0.897691	-0.345	9.77E-05	0.00468
ZNF683	Zinc Finger 683, immune system signaling, regulates memory T, NK, NKT cells	Better survival	**Inhibitor**	257,101	6	1.957393583	0.370498	14	1.003221	1.980712	0.2154	578	− 0.11031	0.896775	− 0.35535	0.001668	0.05578
EP400P1	Ep400 pseudogene 1	Better survival	**Inhibitor**	347,918	6	3.791644858	0.067105	10	1.12437	1.337051	1.49015	582	− 0.02512	1.08331	− 0.18395	0.005293	0.02379
KLHDC7B	Kelch domain containing 7B, <—> cul1, cul3, oncogenic	Poor survival	Enhancer	113,730	6	**0.880909593**	0.80687	30	0.32065	1.37536	0.02375	562	− 0.071	0.905335	− 0.3043	0.003778	0.133445
MT1G	Metallothionein 1G, can inhibit pro-inhibitory cytokines	Poor survival	Enhancer	4495	6	2.477913164	0.218678	4	− 0.44585	0.146572	− 0.46165	588	− 0.03724	0.935006	− 0.32245	0.382064	0.004617

CNA facilitator/suppressor affecting patients’ survival; total 27 genes for which expression levels correlate with both CNA and survival (11 for CNA facilitator, 16 for CNA suppressor). Genes are shown indicating which category/Supplementary Table they are from. After subsequent analysis, nine genes that did not show significance association after adjusting covariates were omitted from Hazard Ratio (HR) calculations. For example, the gene expression of TIGD6 is significantly associated with survival after adjustment of age and stage. But the altered and non-altered group of TIGD6 is not significantly associated with survival after adjustment of age and stage. We also found the gene expression of TIGD6 in altered and non-altered group is not significantly different. The result is interpreted to show that the observed altered status of TIGD6 does not affect/impact its gene expression in TCGA data, but its expression may associate with the survival.

Column G: Highlighted in bold: HR < 1 (for which expression alterations decrease risk). HR > 1 (for which expression alterations increase risk).

The 11 CNA facilitator-survival critical genes were CAPS, CCDC115, ATP6AP1. NBEAP1, SPANXC, TIGD6, C7ORF13, TMEM184A, F8A1, LZTS3, and OLMALINC. Notably, three of these (CAPS/calcyphosin, CCDC115/coiled-coil domain containing 115, ATP6AP1/ATPase + transporting accessory protein 1) are involved in ion transport and/or vacuolar ATPase (V-ATPase), and two (TMEM184A/Transmembrane protein 184A, F8A1/ Coagulation Factor VIII Associated 1) are involved in vesicle transport. Together, these genes suggest a novel survival-critical role of Golgi trafficking in CRC and in CNA management. Two (SPANXC/SPANX family member C, and C7ORF13 [LINC01006]/long intergenic non-protein coding RNA1006) are normally expressed in a testis-specific manner, and their expressions in gastric cancers are associated with EMT, migration, and metastasis^41–43^. TIGD6 (Tigger Transposable Element derived 6) is a DNA-mediated transposon with similarity to a centromere component Cenp B. Based on the Cenp B homology, TIGD6 expression was suspected to interfere with mitotic fidelity and structural integrity of the genome. However, no strong centromere binding of TIGD6-EGFP fusion protein was observed, although binding on the chromosome arms and a low level of binding at centromeres were seen ^44^. Thus, how TIGD6 affects genomic stability currently remains unclear.

The 16 CNA suppressor-survival critical genes were WARS, FOXD4L1, VWA5B2, DDB2, EPOR, ROBO3, PKIB, TMED6, APOBEC3D, B3GNT4, CLCN3, FOXD4, ZNF683, EP400P1, KLHDC7B, and MT1G. Among these, involvement of EPOR (Erythropoietin receptor; involved in JAK2-MAPK/ PI3K/ STAT signaling), DDB2 (Damage specific RNA binding protein 2; involved in UV damage repair and Xeroderma), ROBO3 (Roundabout guidance receptor 3; involved in migration or neurite outgrowth), and MT1G (Metallothionein 1G; involved in protection against oxidative stress and metals) in various cancers is well-documented with hundreds of publications. Three are transcription factors (FOXD4L1; Forkhead Box D4 Like 1, FOXD4; Forkhead Box D4, ZNF683; Zinc Finger Protein 683). Three are transmembrane proteins involved in trafficking (TMED6; Transmembrane p24 trafficking protein 6, B3GNT4; UDP glcNAc betaGal 1,3-N-acetylglucosaminyl transferase 4, CLCN3; Chloride voltage-gated channel 3). Three are immunomodulators (ZNF683, WARS; Tryptophanyl-tRNA synthase1, APOBEC3D; Apolipoprotein B mRNA editing enzyme catalytic subunit 3D). The APOBEC family of enzymes are single-stranded DNA (ssDNA) cytosine-to-uracil (C-to-U) deaminases and are involved in HIV-1 restriction and in mutational generation in cancer. As such, APOBEC enzymes have been proposed as targets for virus and cancer therapy via hypomutation, and small molecule inhibitors are under development^45^. Four are involved in growth regulation (EPOR, PKIB, KLHDC7B, MT1G).

**Figure 4:**
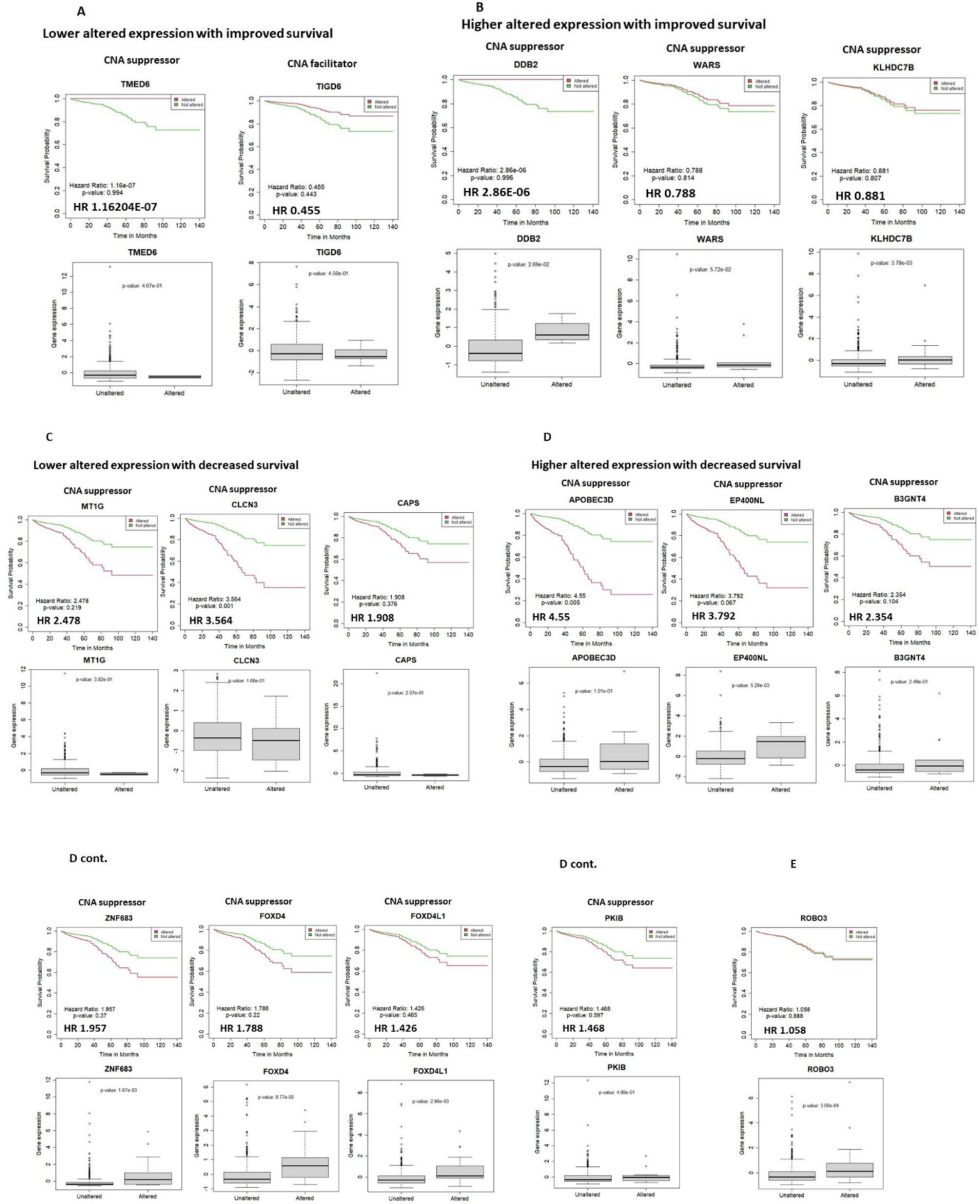
CNA facilitator/suppressor genes affecting patients’ survival (“survival-critical”). For 18 genes, expression levels correlate with both CNA and patients’ survival in CRC (i.e., “survival-critical” genes). The genes represent potential targets for drug development. There are four categories, as follows. (**A**) Lower altered expression with improved survival. For TMED6 and TIDG6, lower expression was associated with improved survival; thus, they are potential inhibitor development targets. Hazard ratio (HR) < 1 (i.e., decreased risk). “Altered” (red), “Not Altered” (green). (**B**) Higher altered expression with improved survival. For DDB2, WARS, and KLHDC7B, higher expression was associated with improved survival; thus, they are potential enhancer development targets. (**C**) Lower altered expression with decreased survival. For MT1G, CLCN3, and CAPS, lower expression was associated with decreased patients’ survival. For HR > 1, expression alterations increase risk. For estimating magnitude of HR, small, medium, and large HRs comparing two groups would be approximately 1.3, 1.9, and 2.8, respectively^34^. (**D**) Higher altered expression with decreased survival. For APOBEC3D, EP400NL, B3GNT4, ZNF683, FOXD4, FOXD4L1, and PKIB, higher expression was associated with decreased survival; thus, they are potential targets for inhibitors. (**E**) ROBO3 is consistently shown to be over-expressed in CRCs. This finding is corroborated by the present study. However, the impact of ROBO3 expression on patients’ survival in CRCs is small (not trivial, but possibly inconsequential) with HR1.058. Figures were generated with cBioportal and with R v4.0.3.

Next, we used tumor data to analyze expression alteration (“altered” vs. “not altered”; definition in Methods section) and hazard ratio (HR), and tested whether expression alteration correlates with survival (see Methods for estimate on HR magnitude^34^. Generally, medium-large HR is > 1.3). The correlations were categorized as (a) lower altered expression with improved survival, (b) higher altered expression with improved survival, (c) lower altered expression with decreased survival, and (d) higher altered expression with decreased survival (Fig. 4). From the standpoint of drug development, developing inhibitor(s) for genes in category (a) or (d) would be most feasible, while developing enhancer(s) of a gene or its function to target categories (b) or (c) remains difficult. For category (a), decreased TIGD6 or TMED6 expression were each associated with improved survival (HR 1.16204E-07 [TMED6], 0.455 [TIGD6]) (Table1; Fig. 4A). For category (b), higher altered expression of DDB2 (HR 2.86E-06), WARS (HR 0.788), or KLHDC7B (HR 0.881) was associated with improved survival (Fig. 4B). As DDB2, WARS, and KLHDC7B are assessed functionally as CNA suppressors, increased expression may be antagonizing high genomic instability. For category (c), decreased MT1G (HR 2.478), CLCN3 (HR 3.564), or CAPS (HR 1.908) expression was associated with poorer survival (Fig. 4C). For category (d), with APOBEC3D (HR 4.55), EP400NL (HR 3.792), B3GNT4 (HR 2.354), ZNF683 (HR 1.957), FOXD4 (HR 1.788), FOXD4L1 (HR 1.426), or PKIB (HR 1.468), higher altered expression was associated with decreased survival (Fig. 4D). On the other hand, ROBO3 is a gene whose overexpression was consistently observed in CRC, and its possible involvement in EMT and malignant progression has been reported^46,47^. Yet, overexpression of ROBO3 showed only small effects on survival in CRCs (HR 1.058). This finding suggests that the amount of ROBO3 expression alone may not be a strong indicator of benefit or disadvantage for survival in CRCs (Fig. 4E). Overall, this analysis identified nine potential target genes (medium-large HR [> 1.3]; TIGD6, TMED6, APOBEC3D, EP400NL, B3GNT4, ZNF683, FOXD4, FOXD4L1, PKIB) for inhibitor development, and four genes (DDB2, MT1G, CLCN3, CAPS) for enhancer development.

## Discussion

At the onset of this project, we anticipated that a similar profile between lung and colon would emerge and a set of genomic instability genes common among cancers would be identified. This expectation was based on (a) pan-cancer analysis of oncogenes that indicated recurring sets of oncogenic pathways common among various cancers (e.g., kras, TP53), and (b) extrapolation from previous pan-cancer analysis of CNA-associated pathways^7^. However, the results were surprising: (a) less involvement of over-expressions of mitotic genes in generating genomic instability in the colon, and (b) the presence of CNA-suppressing pathways, including immune-surveillance, were only partly similar to those in the lung. The results suggest that generation and suppression mechanisms of tumor genomic instability depend on the organ, and that therapeutic modalities targeting genomic instability must be tailored for the target organ.

Although CNA suppression pathways were only partly similar, common to lung and colon were the Antigen Presentation, Interferon Signaling, and Natural Killer Cell Signaling pathways, suggesting the presence of both common/non-organ specific and organ-specific immune components for genomic instability surveillance. This observation may extend to a basis for developing highly organ-specific cancer immuno-prevention or therapies.

This study identified RNA metabolism regulators (e.g., DDX27, PRPF6, SMG5) as influencers of genomic instability in CRC. A mechanistic link between RNA regulators and genomic instability had not been fully explained. Recently, in pancreatic cancer, mRNA regulators/RNA-binding splicing factors were identified as methylation targets of PRMT1 (Protein Arginine Methyl Transferase 1). Inhibition of the methylation via specific inhibitor affects splicing site selection and functional protein expression of the downstream targets. Many of the downstream target proteins, including Cyclin D, were cell cycle and proliferation regulators. Thus, PRMT1 inhibition indirectly caused growth-static effects and genomic instability^48^. We speculate that transcriptomic disturbance of RNA metabolism genes may affect genomic stability in CRC in a similar, indirect mechanism.

Suggesting the validity of this GE-CNA approach, many of the identified pathways are also pathways that have been identified in cancer (chemo) prevention and therapy studies, including apoptosis, Redox signaling, JAK-STAT signaling, and inflammation pathways. The Heme biosynthesis pathway, however, is under-investigated in cancer. As it is newly identified with this unbiased approach, further study is warranted. Regarding MODY signaling, the potential link between diabetes and cancer has been a subject of interest. Meta-analysis indicated that type 2 diabetes (T2D) was associated with incidence of several cancers, especially prostate and liver cancer, and with mortality from pancreatic cancer. In bias analyses, the proportion of studies with a true effect size larger than a RR of 1.1 (i.e., 10% increased risk in individuals with T2D) was nearly 100% for liver, pancreatic, and endometrial cancer; 86% for gallbladder cancer; 67% for kidney cancer; 64% for colon cancer; and 62% for colorectal cancer^49^, indicating a modest level of positive association between CRC and diabetes. However, microsatellite instability was reported to be inversely associated with T2D in CRC^50^. The inverse association between diabetes and MIN-CRC corroborates with our discovery of MODY signaling as suppressor of amplification/insertion CNA, a MIN trait.

Other genes/pathways of interest include APOBEC3 (HR4.6), due to the strong HR, and B3GNT4 (HR2.4), due to its relation to mucin function. APOBEC3D encodes double-domain deaminase and is a member of the APOBEC3 family genes^51^. APOBEC3 proteins form Apolipoprotein B Editing Complex and mediate intrinsic responses to infection by retroviruses [e.g., HIV^52^,], but also can act as a strong mutagenic factor^53^. In breast cancer, expression of APOBEC3B is increased and associated with mutation load and poor outcome, while high APOBEC3C-H expression was linked to favorable prognostic benefit for both cancer progression and mortality^54^. A recent study showed causal relationship between APOBEC3B induction and DNA replication stress and CIN in early breast and lung cancer evolution^55^. Our results with APOBEC3D likely indicate a parallel with APOBEC3B in breast cancer, a mutagenic activity of APOBEC3D in CRCs, and suggest survival benefit with a specific inhibitor of APOBEC3D.

B3GNT4 is a member of the B3GNT family, which is a transmembrane Golgi enzyme that catalyzes the transfer of N-acetyl glucosamine from UDP-GlcNAc onto Gal beta 3 (GlcNAc beta 6) GalNAc-mucin. The enzymes function in the elongation and branching of O-linked oligosaccharide chains of mucin glycoproteins, thus the complete functional maturation of mucins. Mucins play pivotal mucosal barrier functions in the intestine, and their dysfunction is associated with colitis and CRC^56,57^. However, only limited reports portray the importance of mucin maturation enzymes or their value in cancer drug development^58^. B3GNT3 was reported as a novel marker correlated with metastasis and poor clinical outcome in cervical cancer^59^, but to our knowledge this is the first report of potential clinical significance for B3GNT4 in cancers.

Overall, the present study identified genomic instability genes via transcriptomic alterations in CRC, which is an unbiased portrait of genes that may or may not have been identified through previous hypothesis-driven studies. Indeed, this study identified CIN and MIN genes as predicted, as well as a number of genes whose mechanism of generating genomic instability is yet to be investigated. The new results from CRC allows us to compare the profile with that of lung adenocarcinoma. The comparison indicated organ specificity in genes influencing tumor genomic instability and suggests the value of a tailored approach for targeting genomic instability. We identified nine genes whose inhibition may lead to better survival (HR > 1.3; TIGD6, TMED6, APOBEC3D, EP400NL, B3GNT4, ZNF683, FOXD4, FOXD4L1, PKIB) and four genes for which an enhancer may benefit CRC patients’ survival (DDB2, MT1G, CLCN3, CAPS) via genomic instability modulation. These 13 genes with potential clinical relevance carry diverse functions, thus implicating multiple pathways leading to genomic instability rather than single central network affecting genomic instability. With promising target genes identified, further drug development is warranted.

## Supplementary Information

Supplementary Information 1.

Supplementary Information 2.

Supplementary Information 3.

Supplementary Information 4.

Supplementary Information 5.

Supplementary Information 6.

Supplementary Information 7.
