# Supplementary material for: Genomic instability genes in lung and colon adenocarcinoma indicate organ specificity of transcriptomic impact on Copy Number Alterations
Source: Sci Rep. 2022 Jul 11;12:11739. doi: 10.1038/s41598-022-15692-8 (PMC9273645; doi:10.1038/s41598-022-15692-8)
Supplement: Supplementary file 1 — Supplementary Information 1. [file 41598_2022_15692_MOESM1_ESM.docx]

**Legends for Supplementary materials.**

**Table S1: CNA (Copy Number Alterations) facilitator genes.**

This file includes data from the GE-CNA analysis and lists 247 genes whose High expression correlate with high CNA (p-adjusted q value <0.05), thus annotated as CNA facilitator genes.

The data include analysis output on Hugo_Symbol, Entrez_Gene_Id, Average # of CNVs, SD, Average # of CNVs , SD, pvalue, p.adjust.qvalue, HE_increase, p_value, p_value_BH, p_value_qvalue, Approved.symbol, Approved.name, and Alias.symbols.

**Table S2: CNA suppressor genes.**

This file includes data from the GE-CNA analysis and lists 253 genes whose High expression correlate with low CNA (p-adjusted q value <0.05), thus annotated as CNA suppressor genes.

**Table S3: Insertion/amplification CNA facilitator genes.**

This file includes data from the GE-CNA analysis and lists 28 genes whose High expression correlate with high insertion/amplification-type CNA (p-adjusted q value <0.05), thus annotated as Insertion/amplification CNA facilitator genes. As Insertion/amplification CNA is a surrogate marker for Microsatellite Instability MIN, the genes can be considered as MIN facilitator genes.

**Table S4: Deletion CNA facilitator genes.**

This file includes data from the GE-CNA analysis and lists 20 genes whose High expression correlate with high deletion-type CNA (p-adjusted q value <0.05), thus annotated as deletion CNA facilitator genes. As deletion CNA occurs with chromosome instability CIN, the genes can be considered as CIN facilitator genes.

**Table S5: Insertion/amplification CNA suppressor genes.**

This file includes data from the GE-CNA analysis and lists 23 genes whose High expression correlate with low insertion/amplification-type CNA (p-adjusted q value <0.05), thus annotated as Insertion/amplification CNA suppressor genes. The genes can be considered as MIN suppressor genes.

**Table S6: Deletion CNA suppressor genes.**

This file includes data from the GE-CNA analysis and lists 253 genes whose High expression correlate with low deletion-type CNA (p-adjusted q value <0.05), thus annotated as deletion CNA suppressor genes. The genes can be considered as CIN suppressor genes.
